# Dynamics of Inter-heavy Chain Interactions in Human Immunoglobulin G (IgG) Subclasses Studied by Kinetic Fab Arm Exchange[Fn FN1]

**DOI:** 10.1074/jbc.M113.541813

**Published:** 2014-01-14

**Authors:** Theo Rispens, Anna M. Davies, Pleuni Ooijevaar-de Heer, Samira Absalah, Onno Bende, Brian J. Sutton, Gestur Vidarsson, Rob C. Aalberse

**Affiliations:** From ‡ Sanquin Research, 1066 CX Amsterdam, The Netherlands, and Landsteiner Laboratory, Academic Medical Centre, University of Amsterdam, 1105 AZ, The Netherlands,; the § Randall Division of Cell and Molecular Biophysics, King's College London, London SE1 1UL, United Kingdom, and; the ¶ Medical Research Council and Asthma UK Centre in Allergic Mechanisms of Asthma, London SE1 9RT, United Kingdom

**Keywords:** Antibodies, Antibody Engineering, Protein Structure, Structural Biology, Sulfhydryl, Thermodynamics

## Abstract

Interdomain interactions between the CH3 domains of antibody heavy chains are the first step in antibody assembly and are of prime importance for maintaining the native structure of IgG. For human IgG4 it was shown that CH3-CH3 interactions are weak, resulting in the potential for half-molecule exchange (“Fab arm exchange”). Here we systematically investigated non-covalent interchain interactions for CH3 domains in the other human subclasses, including polymorphisms (allotypes), using real-time monitoring of Fab arm exchange with a FRET-based kinetic assay. We identified structural variation between human IgG subclasses and allotypes at three amino acid positions (Lys/Asn-392, Val/Met-397, Lys/Arg-409) to alter the strength of inter-domain interactions by >6 orders of magnitude. Each substitution affected the interactions independent from the other substitutions in terms of affinity, but the enthalpic and entropic contributions were non-additive, suggesting a complex interplay. Allotypic variation in IgG3 resulted in widely different CH3 interaction strengths that were even weaker for IgG3 than for IgG4 in the case of allotype G3m(c3c5*/6,24*), whereas G3m(s*/15*) was equally stable to IgG1. These interactions are sufficiently strong to maintain the structural integrity of IgG1 during its normal life span; for IgG2 and IgG3 the inter-heavy chain disulfide bonds are essential to prevent half-molecule dissociation, whereas the labile hinge disulfide bonds favor half-molecule exchange *in vivo* for IgG4.

## Teaser

**Background:** Fab arm exchange requires weak interactions between CH3 domains, such as in human IgG4.

**Results:** CH3-CH3 interactions differ >1,000,000-fold between human subclasses and allotypes due to variations Lys/Asn-392, Val/Met-397, and Lys/Arg-409.

**Conclusion:** For IgG2 and IgG3, but not IgG1, hinge disulfide bonds are essential to prevent half-molecule dissociation.

**Significance:** Subclass/allotype variation in the CH3 domain can alter antibody stability and functionality.

## Introduction

**FIGURE 1.**
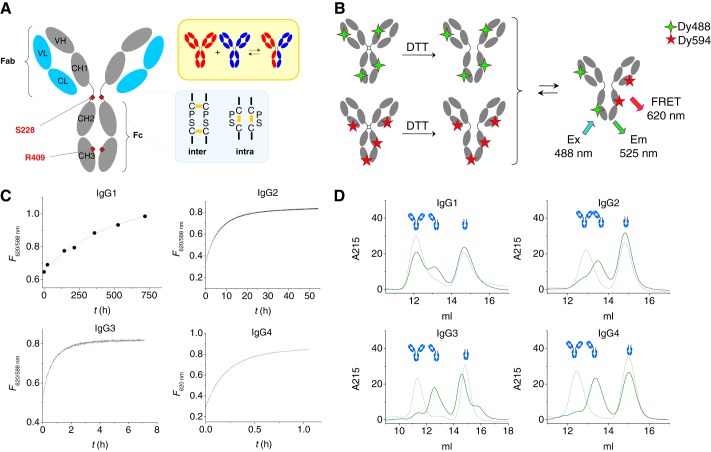
**Fab arm (or half-molecule) exchange reaction of human IgG subclasses with reduced hinge disulfide bonds.** *A*, features in IgG4 that relate to Fab arm exchange. Arg-409 allows for facile dissociation of CH3 domains, whereas Ser-228 introduces flexibility in the hinge facilitating intrachain disulfide bond formation. The *upper right panel* schematically indicates the Fab arm or half-molecule exchange process. *B*, schematic representation of the FRET assay to monitor Fab arm exchange reaction. Antibodies labeled with either DyLight488 or DyLight594 fluorochrome are mixed, and the product of the exchange reaction will contain both fluorescent labels resulting in a FRET signal. *C*, FRET signal monitored in time at 37 °C for equimolar amounts of 488- and 594-labeled human IgG subclasses after reduction by DTT. Note the different time scales. *Red lines* represent fit of a first-order exponential: *k*_obs_ = 5 × 10^−7^, 3 × 10^−5^, 4 × 10^−4^, and 2 × 10^−3^ s^−1^ for IgG1–4, respectively. *D*, IgG and Fc fragments (3-fold molar excess) of IgG1, IgG2, IgG3, or IgG4 were mixed after reduction with DTT and incubated for 24 h at 37 °C (30 days in case of IgG1) before analysis by high performance-size exclusion chromatography (*green lines*). *Black lines* represent *t* = 0 (IgG2, IgG3, and IgG4) or *t* = 7 days (IgG1). A peak representing a structure of ∼100 kDa (at ∼13 ml elution volume) is observed in all cases.

Antibodies are key players in the humoral immune response and are increasingly used as therapeutic agents. Antibodies are involved in the specific recognition of pathogens and can trigger a range of effector mechanisms depending on their exact structural features. Immunoglobulin G (IgG) antibodies, the most abundant class of immunoglobulins, are composed of two heavy and two light chains, each comprising two or four immunoglobulin domains, respectively (Fig. 1*A*). A molecule of IgG contains two Fab arms, which include the first two domains of a heavy chain and an entire light chain. These parts of the molecule contain the complementarity determining regions that are involved in antigen binding. The remaining parts of the two heavy chains form the Fc part, responsible for antibody effector functions, by interacting both covalently in the “hinge region” and non-covalently in their CH3 domains, linking both half-molecules into one antibody molecule. These interactions are therefore of prime importance for the folding, structural integrity, and functionality of the molecule. In addition, the CH2 domains contain *N*-linked glycans that are important for the correct folding of the Fc part and influence Fcγ receptor binding and complement activation (37, 42).

Human IgG4 is a striking example of altered functionality as a consequence of altered inter-heavy chain interactions. Due to relatively labile disulfide bonds in the hinge as well as weak CH3-CH3 interactions, IgG4 is able to engage in Fab arm exchange (1–4); although regular IgG antibodies are bivalent, IgG4 becomes effectively monovalent by exchanging half-molecules with other molecules of IgG4, combining random specificities in the second Fab arm for a given specificity of the first Fab arm (Fig. 1*A*). Consequently, these antibodies are unable to cross-link antigen to form immune complexes. This process has been observed only for IgG4 and not for IgG1; IgG2 and IgG3 have not been examined thus far. A single amino acid difference between the CH3 domains of IgG1 (Lys-409) and IgG4 (Arg-409) was found to be responsible for facile dissociation of the latter compared with no observable dissociation of the former (3–5). Furthermore, a serine rather than a proline at position 228 in the core hinge of IgG4 introduces flexibility, allowing formation of “intrachain“ disulfide bonds (6, 7) that result in loss of covalent interactions between the heavy chains (Fig. 1*A*).

The stability and dimerization of CH3 domains has been addressed in a number of studies that showed folding to precede dimerization and demonstrated a role for the intradomain disulfide bond in stabilizing the interdomain interactions (8, 9). Dissociated mouse CH3 monomer was found to be only short-lived (10), and single-molecule force spectroscopy experiments revealed the CH3-CH3 dimer to be extremely stable compared with other multimers studied by the same technique, indicating that CH3-CH3 interactions in general may be very strong. For human IgG1, this interaction has been estimated to have an upper limit for the *K_d_* of ∼10^−10^ m (11, 12). Whereas a reasonable estimate of the strength of the CH3 interactions for IgG4 has been obtained in several studies (*K_d_* between 2 and 4 nm) (3–5), such data are lacking for the other subclasses. Indirect evidence suggests that the interactions between heavy chains may be weaker in the case of IgG2 compared with IgG1 (13–15), suggesting that subclass-specific determinants other than Lys/Arg-409 can affect the CH3 dimerization strength.

**FIGURE 2.**
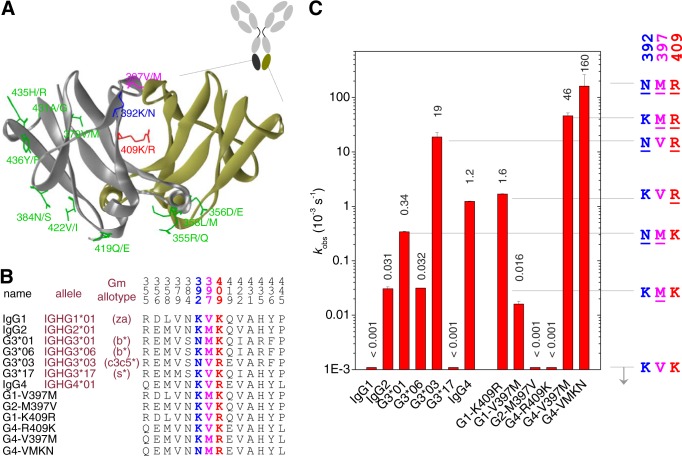
**Subclass and allotype differences that influence the rate of CH3-CH3 dissociation.** *A*, crystal structure of an IgG4 CH3 dimer (PDB code 4B53) (55). Indicated are all amino acid positions for which variation exists between human IgG subclasses or allotypes. *B*, listed are the Fc constructs used in this study with the amino acids at these respective positions. For each subclass, the canonical structure has an allele name ending in *01, *e.g.* IGHG1*01 for IgG1 (30). For IgG3, an additional three allelic forms were also examined. Full sequences are listed in supplemental Fig. S2. *C*, observed rate constants at 37 °C for the half-molecule exchange of Fc constructs (with reduced hinge disulfide bonds) as measured in the FRET assay.

Key elements of the CH3 dimerization interface were identified for human IgG1, in particular amino acid positions Thr-366, Leu-368, Phe-405, Tyr-407, and Lys-409, which make up the center of the interface and are conserved among all IgG subclasses with the notable exception of Lys-409 (which is Arg-409 in IgG4) (2, 3, 5, 11, 16). However, the edges of the interface include amino acids that differ between IgG subclasses (Fig. 2*A*). Moreover, besides structural variation between the IgG subclasses that potentially affects the stability, polymorphisms also result in structural variation within human IgG subclasses. Human IgG3 is particularly polymorphic, and a number of unique, serologically recognizable determinants (allotypes) have been mapped to specific amino acid substitutions in the CH3 domains (17–19). Interestingly, one of the polymorphisms in IgG3 that has not been mapped to any serological allotypic marker (Lys/Asn-392) is located at the edge of the CH3-CH3 dimerization interface, thus raising the possibility that this polymorphism may alter the stability of these interactions.

We recently developed a method to monitor the kinetics of Fab arm exchange of IgG4 half-molecules in real time by monitoring the appearance of a Förster resonance energy transfer (FRET) signal upon recombination of IgG4 half-molecules containing two different fluorochromes (4). Real-time monitoring of Fab arm exchange allows accurate measurement of the CH3-CH3 dissociation rate, providing insight in the dynamics of inter-heavy chain interactions in human IgG in its native form. Here, we systematically investigated non-covalent inter-heavy chain interactions for the different human subclasses as well as polymorphisms in the CH3 domains. We found these interactions to vary in strength by more than 6 orders of magnitude, which sheds new light on the role of CH3-CH3 interactions in maintaining inter-heavy chain interactions and modulating Fab arm exchange.

## EXPERIMENTAL PROCEDURES

### 

#### 

##### Materials

Recombinant chimeric IgG1 and IgG4 antibodies and mutants (against *Fel d 1*) were produced as described previously (2, 13); Fc fragments of IgG1 and IgG4 were obtained as described (2, 20). Recombinant chimeric IgG2 and IgG3 antibodies (against biotin) were produced essentially as described before (21) by cloning synthetic constructs coding for the variable domains (22, 23) and IgG2, IgG3, and κ constant domains (obtained from GeneArt; Invitrogen) into a pcDNA3.1 expression vector (Invitrogen). Other monoclonal IgG1, IgG2, and IgG4 antibodies used were adalimumab (Humira, Abbott), panitumumab (Vectibix, Amgen), and natalizumab (Tysabri, Biogen Idec, Inc.).

Fc fragments of different subclasses and allotypes were produced (CH2CH3 with an IgG4 hinge) by cloning synthetic constructs (obtained from GeneArt; Invitrogen) into a pcDNA3.1 expression vector (Invitrogen) essentially as described before (24). Expression vectors were used for transient transfection of HEK293F cells with 293fectin and Opti-MEM (Invitrogen) using the Freestyle HEK293F expression system (Invitrogen) according to the instructions supplied by the manufacturer. Cell culture supernatants were centrifuged for 15 min at 1700 × *g* followed by loading on a Protein G column (Protein G4 fast flow; GE Healthcare) and elution of the Fc constructs with 0.1 m glycine, pH 2.5–3. The eluate was neutralized immediately with 2 m Tris-HCl, pH 9, and dialyzed overnight to PBS. After dialysis, samples were stored at −20 °C. Concentrations were determined by *A*_280_ measurement using a Nanodrop ND1000.

##### A Note on Allotypes

Polymorphisms in the IgG genes lead to structural determinants, many of which were originally found by serologic methods (17–19, 25, 26, 28). In this paper we used the more convenient alphabetical notation to designate the various allotypes (17–19). For IgG1, polymorphism in the CH3 domain results in the G1m(a) allotype (Asp-356/Leu-358) or the nG1m(a) isoallotype (Glu-356/Met-358). Many polymorphisms are known for IgG3 (29). The allotypic determinants that can be distinguished by specific antibodies have been mapped to particular amino acids (17–19). Sequences of the different alleles can be found on the IMGT website; the canonical structure of each subclass has an allele name ending in *01, *e.g.* IGHG1*01 for IgG1, which corresponds to G1m(za). In this paper we examined several common IgG3 allelic structures: G3m(b*), G3m(c3c5*), and G3m(s*), where the shorthand nomenclature is: b* = b0, b1, b3, b4, b5, u, v; c3c5* = b0, b1, c3, u, c5; s* = b0, b3, b5, s, v (30). For G3m(b*) structure, there is additional genetic variation not captured in the allotype nomenclature system, including variation at position 392. We examined two allelic forms of G3m(b*), *i.e.* IGHG3*01 and IGHG3*06. Complete sequences, allele names, and IMGT accession numbers for all structures are provided in supplemental Figs. S1 and S2.

##### Fluorescent Labeling

Antibodies and antibody fragments were fluorescently labeled with DyLight 488 or DyLight 594 amine reactive dye (Pierce/Thermo Scientific) according to the instructions of the manufacturer. Unreacted dye was removed by repeated dilution/concentration using Amicon Centriprep centrifugal filter devices (Millipore, Billerica, MA) until no dye could be detected anymore in the filtrate. The average degree of labeling was between 4 and 6 for IgG antibodies and ∼2 for Fc fragments.

##### Kinetic Measurements (FRET)

Kinetics of the exchange reactions were monitored in real-time using a previously developed FRET assay (4). Briefly, the reactions were carried out using one of the following protocols. A) 4 μg/ml Fc-488 and Fc-594 in PBS-P (degassed PBS containing 0.05% poloxamer 407 (Lutrol F127; BASF)) were incubated separately for 1 h at 37 °C in the presence of 3 mm dithiothreitol (DTT). Then the samples were incubated at the required temperature (10–37 °C), and the reaction was initiated by mixing equal volumes of both solutions into a thermostatted quartz cuvette. For IgG4-Fc-V397M/K392N, reactions were carried out at 10 μg/ml final concentration. B) 2 μg/ml IgG-488 and IgG-594 were mixed and equilibrated at 37 °C. Then the reaction was initiated by adding several microliters of a concentrated stock solution of GSH to a final concentration between 0.25 and 10 mm. Kinetics were monitored by measuring the appearance of a FRET signal using a Varian Cary Eclipse fluorescence spectrophotometer equipped with a thermostatted multi-cell holder (excitation, 488 nm; emission, 620 nm; emission at 588 nm was used to account for base-line drifts during slower reactions, 588 nm being the isosbestic point in the overlay spectrum). To compare experimental rate profiles, normalized fluorescence was calculated as *F*(norm.) = (*F_t_* − *F*_0_)/(*F*_max_ − *F*_0_), where *F_t_* is fluorescence at time *t, F*_0_ is fluorescence at *t* = 0, and *F*_max_ is the end-point fluorescence. The rate of IgG4 Fab arm exchange was also measured in a redox buffer that mimics plasma (31, 32): 0.14 μm oxidized glutathione (GSSG), 3 μm reduced glutathione (GSH), 0.01 mm reduced cysteine (Cys), and 0.04 mm oxidized cysteine (CySS) in PBS. Activation parameters were obtained by measuring the dissociation rates at 4–5 different temperatures between 10 and 37 °C as described above; fitting a straight line to a plot of ln(*k*_obs_*h*/*k_b_T*) *versus* 1/*T* (Eyring plot) yields the activation enthalpy (slope = −*R* Δ*H*) and entropy (intercept = −*R* Δ*S*) (33).

##### High Performance-Size Exclusion Chromatography

IgG1, IgG2, IgG3, and IgG4 were mixed with the respective IgG Fc and incubated with 10 mm DTT in degassed PBS. Final concentrations of both IgG and IgG Fc were 20 μg/ml. The mixtures were incubated at 37 °C for 30 days (IgG1), 7 days (IgG2), and 24 h (IgG3, IgG4) and quenched by adding a 2-fold molar excess of iodoacetamide compared with DTT. Samples were stored at −20 °C until they were analyzed by applying 50 μl to a Superdex 200 HR 10/300 column (Amersham Biosciences), which was connected to a HPLC system (ÄKTAexplorer) from Amersham Biosciences. The column was equilibrated in PBS. Elution profiles were monitored by measuring UV absorption at 214 nm.

##### Equilibrium Constants (Fluorescence-assisted High-performance Liquid Chromatography)

To determine equilibrium constants, IgG2, G3*01, IgG4, and IgG1-K409R and DyLight-488 labeled equivalents were reduced with 10 mm DTT (60 min/37 °C) and alkylated with 22 mm iodoacetamide. Serial 4-fold dilutions of reduced/alkylated IgG (0.001–300 nm half-molecules) were incubated with 0.05 ng/ml reduced/alkylated IgG2–488, 0.2 ng/ml IgG3, or 1 ng/ml IgG4/IgG1-K409R in PBS containing as a carrier protein 0.1 mg/ml certolizumab pegol, and 0.02% Tween 20 and incubated at 37 °C for up to 3 days before analysis. Between 50 and 1000 μl of a sample was applied using an autosampler to a Superdex 200 HR 10/300 column that was connected to an ÄKTAexplorer HPLC and eluted at 0.5 ml/min. Elution profiles were monitored by measuring the fluorescence (excitation/emission 488/525 nm) with a Prominence RF-20Axs in-line fluorescence detector (Shimadzu, Kyoto, Japan). Dissociation constants were calculated as described before (34).

##### Equilibrium Constants (FRET)

Equilibrium constants of G3*03 Fc, IgG4-V397M Fc, and IgG4-V397M/K392N Fc were determined by recording fluorescence spectra using either a Varian Cary Eclipse fluorescence spectrophotometer or a Nanodrop ND3300 of 2-fold dilutions of equimolar mixtures of Fc-488 and Fc-594 after incubation for 2 h at 37 °C in the presence of 3 mm DTT. IgG4 was also included to compare both methods to determine *K_d_*. Spectra were corrected for background fluorescence, and the ratio of *F*_620_/*F*_588_ was plotted against total concentrations of half-molecules (A+B) to obtain a dose-response curve (4). The concentration of mixed IgG4 dimers (AB) will correlate linearly with the total amount of IgG4 dimers (A_2_ + B_2_ + AB); therefore, the FRET signal is representative of the amount of dimers present in solution. The dissociation constant was calculated by fitting a homodimerization model to the data (see above).

##### SDS-PAGE

Non-reducing SDS-PAGE was performed according to the instructions of the manufacturer (Invitrogen) using 4–12% gradient gels. Samples were heated for 10 min at 70 °C. Iodoacetamide was added to the sample buffer to minimize artifacts (35). Gels were stained with PageBlue staining solution (Thermo Fischer, Waltham, MA).

##### Modeling

Protein three-dimensional structures were visualized using Discovery Studio 3.5, Accelrys.

##### Structural Analysis of the CH3-CH3 Interface

To assess the potential structural effects of the V397M and K392N substitutions at the CH3-CH3 interface, crystal structures for human IgG1-Fc (36–51) and PDB codes 3DO3,^3^ 3D6G,^4^ and 1OQO and 1OQX,^5^ IgG2-Fc (52, 53), IgG4-Fc (54), and the IgG4 CH3 domain (55), all solved at 2.6 Å resolution or higher, were analyzed using Coot (56). For the V397M substitution, structures for IgG1 and IgG4 (V397) were compared with those for IgG2 (Met-397). On the other hand, because only structures with Lys-392 have been solved thus far, for the K392N substitution, structures were examined so that the nature of a typical CH3-CH3 interface in the vicinity of Lys-392 could be established. Lys-392 was then mutated to asparagine in one IgG1-Fc structure with Coot (PDB code 3AVE) (51), and potential effects of different N392 rotamer positions were investigated.

## RESULTS

### 

#### 

##### CH3-CH3 Interactions in Human IgG Subclasses

We investigated the rate of dissociation of CH3 domains for all four human IgG subclasses using a previously developed FRET assay to monitor Fab arm exchange in real time (Fig. 1*B*). If no interheavy chain disulfide bonds were present, the CH3-CH3 dissociation was the rate-determining step of the Fab arm exchange, allowing for direct quantification of the CH3-CH3 dissociation rate (4). Removal of Fc glycans did not affect the observed rates (not shown). DyLight-488- and DyLight-594-labeled antibodies were reduced with DTT resulting in a loss of covalent interactions between the heavy chains. Equimolar amounts of the reduced antibodies were then mixed, resulting in Fab arm exchange for all subclasses but at vastly different rates (Fig. 1*C*). To confirm that an exchange reaction was indeed taking place, antibodies were also incubated with Fc fragments of the respective subclass, and in all cases a 100-kDa structure corresponding to the product of the exchange reaction was observed (Fig. 1*D*). For IgG1, the dissociation rate was very low (*k*_obs_ ≈ 5 × 10^−7^ s^−1^), an almost 5000-fold difference compared with IgG4 (*k*_obs_ = 1.8 × 10^−3^ s^−1^). On the other hand, the dissociation rates for IgG2 and IgG3 were 60- and 4-fold lower compared with IgG4, respectively. It appears that structural variation among the subclasses other than Lys/Arg-409 influences the interaction strength between the CH3 domains. Moreover, the intrinsic half-life for dissociation of the CH3 domains was much shorter for all subclasses except IgG1, *i.e. t*_½_ = 16 days, 6.5 h, 28 min, and 6 min for the dissociation of IgG1, 2, 3, and 4, respectively, compared with their *in vivo* half-life of 21 days (7 days for IgG3). These results imply that the inter-heavy chain disulfide bonds of IgG2 and IgG3 will play a crucial role in determining their stability and raise the question if Fab arm exchange might be observed under conditions that mimic the *in vivo* redox conditions.

##### Subclass-specific Amino Acids That Affect CH3-CH3 Interactions

Next, we systematically investigated the role of subclass-specific determinants and immunoglobulin polymorphisms that may influence the strength of the CH3-CH3 interactions. The sequence of the CH3 domains of all known polymorphic variants of human IgG subclasses were aligned (supplemental Fig. S1), and all amino acid positions that differ between any two sequences are highlighted in a three-dimensional model of a CH3-CH3 dimer (Fig. 2, *A* and *B*). Three of these reside at the CH3-CH3 interface: position 392 (Lys in most sequences but Asn in some IgG3 allotypes), position 397 (Val for IgG1/4, Met for IgG2, and Val or Met for IgG3, depending on the allotype), and position 409 (Lys for all subclasses except IgG4 and G3m(c3c5*), where it is Arg). In addition, amino acids at positions 356 and 358 (Asp and Leu for the G1m(a) allotype, and Glu and Met for the nG1m(a) isoallotype) might influence the CH3-CH3 interactions due to their close proximity to the CH3-CH3 interface. Based on these differences, we prepared a panel of Fc fragments that covers the variations in the CH3 interface between human IgG subclasses and allotypes. The panel includes four different allelic forms of IgG3 and several single-point mutants at positions 392, 397, and 409 (Fig. 2*B*; supplemental Fig. S2). All Fc fragments contained an IgG4 core hinge, were fluorescently labeled, and were reduced by DTT to determine the dissociation rate at 37 °C using the FRET assay described above.

##### IgG4; Arg-409

Introducing a K409R mutation in IgG1 (G1-K409R), a situation found in IgG4 (and some IgG3 allotypes, see below), results in a structure with an increased exchange rate, that is equal to that of IgG4 (Fig. 2*C*), suggesting that this particular amino acid difference between IgG1 and IgG4 is the sole determinant that makes the CH3 interactions in IgG1 “IgG4-like.” It also implies that the structural variation at positions 356 and 358 (*i.e.* “a” *versus* “non-a” allotype) does not result in an appreciable difference in stability.

##### IgG2: Met-397

Comparing IgG1 and IgG2, the only structural difference in the CH3-CH3 interface is found at position 397. Indeed, the dissociation rate for the G1-V397M mutant was similar to IgG2 (Fig. 2*C*). Conversely, no exchange was observed in this experiment for the G2-M397V mutant (*k*_obs_ < 1 × 10^−6^ s^−1^). Thus, a methionine at position 397 significantly destabilizes the interactions between the CH3 domains, albeit to a smaller extent than an arginine at position 409.

##### IgG3: Asn-392, Met-397, and Arg-409

The IgG3 used in Fig. 1 corresponds to IGHG3*01, and like IgG2 also contains a methionine at position 397 but also an asparagine at position 392. Interestingly, a polymorphic variation exists (IGHG3*06) differing only at position 392, with a lysine like the other isotypes, thus resembling IgG2 at all position in the CH3-CH3 interface. Accordingly, IgG2 and G3*06 were found to indeed dissociate at equal rates (Fig. 2*C*). However, G3*01 dissociates >10-fold faster compared with G3*06, demonstrating that an asparagine at position 392 results in weaker CH3-CH3 interactions leading to the relatively fast exchange reaction for this IgG3 allotype (Figs. 1*C* and 2*C*).

Interestingly, another IgG3 allotype, IGHG3*03 or G3m(c3c5*), resembles IgG4 in having an arginine at position 409 but also has the asparagine at position 392. In line with the abovementioned observations, G3*03 was found to dissociate ∼15-fold faster than IgG4, making it the least stable naturally occurring IgG3 allotype.

Yet another IgG3 allotype, IGHG3*17 or G3m(s*), with identical residues as found in IgG1 at all the three positions differing between the subclasses in the CH3-CH3 interface, which were found here to influence the stability of the CH3-CH3 interactions, *i.e.* Lys-392, Val-397, Lys-409, was as stable as IgG1, confirming the importance of these three key amino acids in explaining the differential stability of the CH3-CH3 interactions between IgG subclasses and allotypes thereof.

##### Combined Effects of Asn-392, Met-397, and Arg-409

The combined effects of the destabilizing mutations at positions 392 and 397 in combination with Arg-409 in the backbone of IgG4 were also tested. Introducing a V397M mutation, found in IgG2 and some IgG3 allotypes, into IgG4 resulted in a labile structure that easily dissociates. Additionally, introducing a K392N mutation, found in some IgG3 allotypes, caused a further destabilization of the CH3-CH3 interactions, and a *k*_obs_ = 1.6 × 10^−1^ s^−1^ was found nearly 6 orders of magnitude faster compared with IgG1 and >100 times faster than wild-type IgG4. In summary, we identified two new positions in the CH3 domains of IgG where amino acid variations as present among human IgG subclasses and IgG allotypes resulted in altered stability: Lys/Asn at position 392 and Val/Met at position 397.

##### Interactions between IgG1 CH3 Domains Are Exceptionally Strong

**FIGURE 3.**
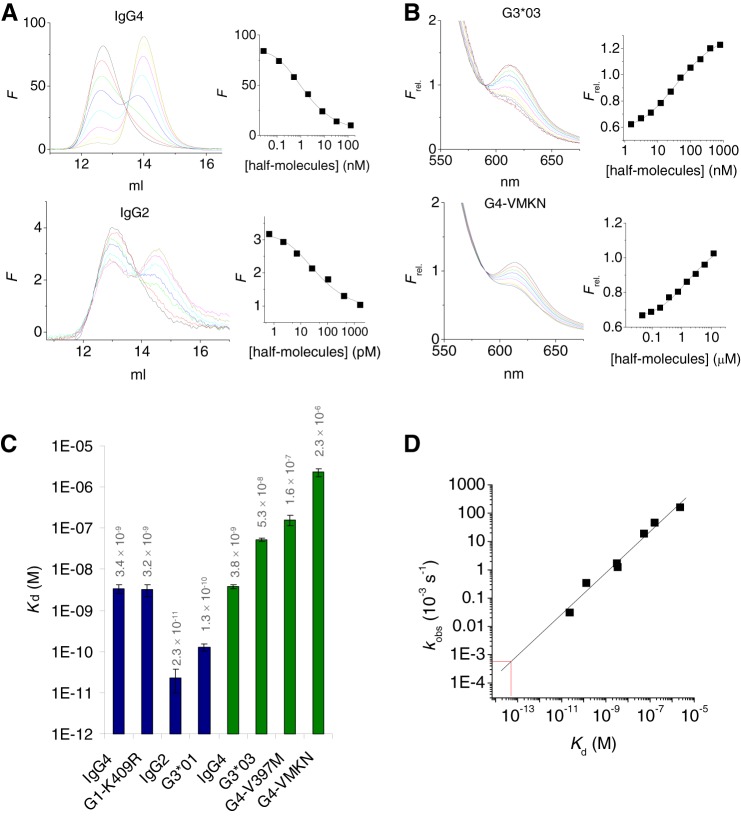
**Strength of CH3-CH3 interactions.** *A*, a small, fixed dose of DyLight488-labeled antibody (1 and 0.05 ng/ml for IgG4 and IgG2, respectively) was incubated with different concentrations of unlabeled antibody at 37 °C in the presence of DTT, and samples are analyzed by high performance-size exclusion chromatography (*left panels*). *Right panels*, fluorescence at 14.5 ml (representing dissociated half-molecules) was plotted *versus* the total concentration of half-molecules. The *solid line* represents a fit of a 1:1 dissociation model to the data. *B*, equimolar amounts of DyLight488 and DyLight594-lableled Fc fragments are incubated at various concentrations in the presence of DTT and fluorescence spectra recorded. *Right panels*, fluorescence at 620 nm (FRET signal; relative to the fluorescence at 588 nm, the isosbestic point; Ref. 4) plotted *versus* the total concentration of half-molecules. The *solid line* represents a fit of a 1:1 dissociation model to the data. *C*, dissociation constants as determined by HPLC (*blue bars*) or FRET (*green bars*). For IgG4, both methods yielded an essentially equal *K_d_. D*, observed rate constants (Fig. 2*C*) representing the dissociation reaction correlate well with the dissociation constants *K_d_* (*r*^2^ = 0.98, slope 0.73). *Red lines* indicate the estimate of the *K_d_* for IgG1 based on the dissociation rate constant.

To learn more about the CH3-CH3 interactions, we also measured the *K_d_* values for a number of the Fc constructs (Fig. 3). For the stronger interactions, we used our recently developed fluorescently assisted HPLC binding assay (34). For the weaker interactions we determined the dissociation constants by monitoring the FRET signal as function of the concentration as we did before for IgG4 (Fig. 3, *A* and *B*) (4). The results are summarized in Fig. 3*C*. The dissociation constants that were measured span 5 orders of magnitude: a *K_d_* of 23 pm was found for IgG2, whereas for G4-VMKN a value of 2.3 μm was obtained. Notably, a plot of *k*_obs_ *versus K_d_* shows a good linear correlation (*r*^2^ = 0.98) and a slope of 0.73. In other words, the variation in stability is dominated by variation in the dissociation rate and much less by variation in the association rate. For IgG1 no dissociation constant could be measured. However, a reasonable estimate was obtained by extrapolating the linear trend between *K_d_* and *k*_obs_; the dissociation rate measured for IgG1 translates to a *K_d_* between 10^−13^ and 10^−14^ M, which implies that the interdomain interactions between the CH3 domains are exceptionally strong for IgG1.

**FIGURE 4.**
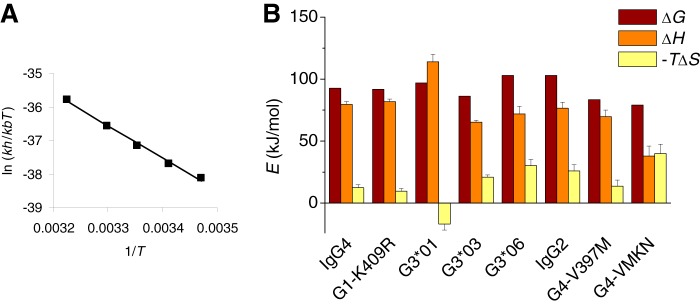
**Activation parameters for the CH3-CH3 dissociation of Fc fragments demonstrate non-additive entropic and enthalpic contributions in the variations in interaction strengths.** *A*, example of an Eyring plot for IgG4 Fc derived from kinetic measurements between 15 and 37 °C. *B*, activation parameters at 25 °C for different Fc fragments. Further details are provided under “Experimental Procedures.”

Additional insight about the effects of the different mutations can be obtained from activation parameters (Δ*G*, Δ*H*, Δ*S*). Although the dissociation rates suggest that the contributions of mutations at these three positions are essentially additive, activation parameters demonstrate substantial non-additive contributions in the enthalpy and entropy of activation (Fig. 4). For instance, K392N results in opposite enthalpic and entropic contributions when introduced in IgG3 (G3*06 *versus* G3*01) or in IgG4 (IgG4 *versus* G4-K392N). The interactions of these amino acids apparently mutually affect each other. The enthalpic contributions dominate the dissociation process and, therefore, probably also the interaction strength.

##### The Importance of Hinge Disulfide Bonds

Because we found that IgG molecules dissociate relatively fast into half-molecules in the absence of disulfide bonds (with the exception of IgG1), it suggests the hinge disulfide bonds are even more important for maintaining structural integrity of antibody molecules than previously recognized, and even transient reduction may result in Fab arm exchange. Therefore, we also investigated the susceptibility toward Fab arm exchange for different hinge configurations at different redox conditions. Two “IgG1-like” hinge configurations were examined: a “hinge-stabilized” version of IgG4 bearing an S228P mutation (a mutation that is present in a number of therapeutic IgG4 antibodies currently in development) as well as IgG1-K409R and two IgG4-like structures, IgG4, and IgG1-K409R/P228S, abbreviated as IgG1-KRPS. The latter IgG1 mutant contains both features that are unique to IgG4 and allow facile Fab arm exchange.

**FIGURE 5.**
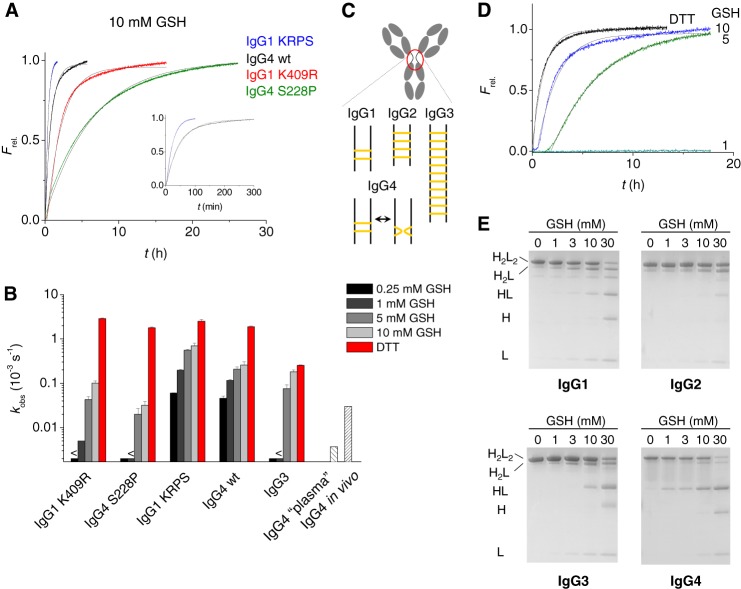
**Fab arm exchange reaction for full-length antibodies with or without stabilized hinge.** *A*, examples of rate profiles at 10 mm GSH and 37 °C. The profiles could be reasonably well approximated by a single exponential indicating a fast pre-equilibrium between covalent and noncovalent isomers (4) (*fitted lines in gray*). *B*, observed rate constants at different redox conditions (37 °C). For IgG3 (IGHG3*01), these values are approximations that do not fully reflect the rate profiles and neglect the lag phase as shown in *D*. For IgG4, additionally shown are observed rates of Fab arm exchange in a redox buffer that mimics the plasma redox conditions as well as the approximate *in vivo* rate constant based on animal models (2, 3, 16). *C*, schematic overview of inter-heavy chain disulfide bonds in IgG subclasses. *D*, rate profiles for the exchange reaction of IgG3 (IGHG3*01) at different redox conditions (37 °C). *E*, non-reducing SDS-PAGE of IgG1–4 after incubation for 24 h at different concentrations of GSH. KRPS, K409R/P228S.

If the disulfide bonds are fully reduced, the rate of Fab arm exchange was similar (less than a factor of 1.5 different) for wt IgG4, its S228P mutant, and for both IgG1 mutants (Fig. 5). However, mildly reducing conditions resulted in markedly different exchange rates. Both IgG4-like hinge configurations demonstrated a modest variation in rate depending on the concentration of reduced glutathione (GSH). At concentrations as low as 0.25 mm, the rate is only a factor of 5–10 lower compared with 10 mm GSH. On the other hand, the IgG1-like hinges do not participate in observable Fab arm exchange at 0.25 mm GSH, and a slow Fab arm exchange becomes noticeable only at concentrations of GSH between 1 and 5 mm. This much steeper dependence on the GSH concentration reflects a different mechanism of Fab arm exchange (see “Discussion”). Neither IgG1-K409R or IgG4-S228P was found to exchange *in vivo* in animal models (57, 58). The differential susceptibility for Fab arm exchange at low concentrations of GSH may reflect the ability to participate in *in vivo* Fab arm exchange. Thus, an appreciable exchange reaction at concentrations of GSH below 1 mm might be indicative for *in vivo* Fab arm exchange. In line with this hypothesis, the rate of exchange at concentrations of GSH between 0.25 and 1 mm matches the reported values of *in vivo* Fab arm exchange for IgG4 (2, 3, 16). Nevertheless, such concentrations of GSH are still well above those measured in plasma, and even the most abundant reductant in plasma, cysteine, is only present at 0.01 mm (31, 32). Fab arm exchange of IgG4 was still observed in a redox buffer that mimics plasma but was an order of magnitude slower than the actual rate of exchange calculated from *in vivo* data (Fig. 5*B*) (2, 3, 16).

For IgG3, with its extended and multiple disulfide-linked hinge (Fig. 5*C*), it was especially relevant to examine the susceptibility for Fab arm exchange at mildly reducing conditions. At 0.25 or 1 mm GSH no Fab arm exchange was observed, which suggests that the propensity to participate in Fab arm exchange *in vivo* is probably low (Fig. 5, *B* and *D*). However, at 5 mm GSH Fab arm exchange was efficient, and the approximate rate of exchange was ∼3-fold lower compared with the situation in which the disulfide bonds were fully reduced by DTT. The steep concentration dependence suggests a cooperative reduction of disulfide bonds.

The formation of noncovalent hinge isomers was also examined at different concentrations of GSH for all subclasses by SDS-PAGE (Fig. 5*E*). The variation of the rate of exchange with concentration of GSH is reflected in the appearance of noncovalent isomers, demonstrating that breaking of disulfide bonds, or in the case of IgG4, the formation of intrachain hinge isomers controls if, and how fast Fab arm exchange may take place.

##### Structural Analysis of V397M and K392N Substitutions

**FIGURE 6.**
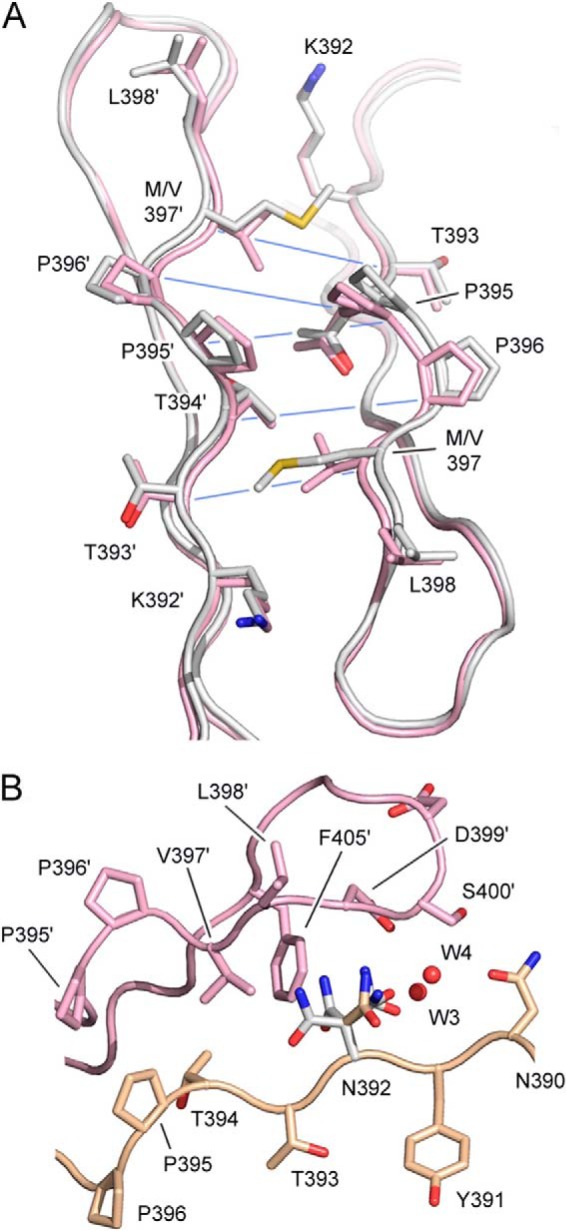
**Effects of Met-397 and Asn-392 on the CH3-CH3 interface.** *A*, the IgG1 (*pink*) and IgG2 (*white*) CH3-CH3 interface in the vicinity of residue 397, which is valine in IgG1 and methionine in IgG2. Cα-Cα distances, which are increased due to the presence of Met-397, are indicated with a *blue line*. PDB codes are 3AVE (51) and 4L4J (53), respectively. *B*, the IgG1 CH3-CH3 interface. Residue 392 is lysine in IgG1 but has been mutated to asparagine using an IgG1-Fc structure as a template (PDB code 3AVE). Asn-392 conformers colored in *white* are those that clash with Val-397′, Phe-405′, Asp-399′, and a conserved water molecule (W4). The conformer, colored in *gold*, is accommodated at the CH3-CH3 interface without any clashes and could form a hydrogen bond with Asp-399′ and surface water molecules (not shown). Carbon atoms from the two domains are colored in *pink* and *gold*.

For IgG4 it has been described that Arg-409 disrupts a network of water-mediated hydrogen bonding that is conserved in IgG1, which explains the weaker CH3-CH3 interactions (54, 55). Here, crystal structures of IgG1, IgG2, and IgG4 Fc were examined to rationalize the altered CH3-CH3 interactions upon V397M and K392N substitutions. Residue 397 is located on the D-strand of the CH3 domain, and its side chain is oriented toward the Thr-393′ main chain (the prime denotes residues from the second CH3 domain). Residues in the immediate vicinity of residue 397, whether through the side chain or main chain contacts, are Pro-396, Leu-398, Asp-399, Phe-405, Lys-392′, Thr-393, Thr-394′, and Pro-395′ (Fig. 6*A*). In IgG1 and IgG4, Cα-Cα atom distances in the vicinity of Val-397 are essentially identical, except for those from the DE loop, which are affected by Lys/Arg-409 variation (54, 55). In IgG2, the presence of Met-397 rather than Val-397 causes an increase in the average Cα-Cα atom distance for nearby residues, with the greatest increase between Pro-395 and Pro-395′ (7.8–8.9 Å) followed by Pro-396–Thr-394′ and Met-397–Thr-393′ (8.1–8.8 and 8.0–8.7Å, respectively), and smaller increases for Met-397–Lys-392′ and Phe-405–Thr-393′ (8.0–8.4 and 10.1–10.4 Å, respectively) (Fig. 6*A*). The Leu-398–Lys-392′ and Asp-399–Lys-392′ Cα-Cα distances are not affected by the V397M substitution. The effects of Met-397 on the CH3-CH3 interface are thus local in nature, and the average buried surface area for the IgG2-Fc CH3-CH3 interface (2319 Å^2^) is comparable with the IgG1 average (2313 Å^2^).

Residue 392 is also located on the D-strand of the CH3 domain, close to residue 409. Although the Lys-392 side chain does adopt slightly different conformations, these all point away from the interface. The effects of Lys/Asn-392 variation was assessed by simulating mutation of Lys-392 to Asn-392 in one crystal structure of IgG-Fc (Fig. 6*B*). It was found that some Asn-392 conformers could form hydrogen bonds with the Asp-399′ side chain and Leu-398′ main chain as well as surface water molecules. However, other conformers clashed with Phe-405′ and Asp-399′ side chains. Additionally, one Asn-392 conformer could disrupt a conserved water molecule network at the CH3-CH3 interface (W1–4) in a similar manner to Arg-409 in IgG4 (54). In IgG4, one Arg-409 conformer disrupts binding of W2, whereas Asn-392 could disrupt binding of W4.

## DISCUSSION

Using real-time monitoring of Fab arm exchange, we systematically studied the interactions between the heavy chains in human IgG subclasses. The stability of the inter-heavy chain interactions differs >6 orders of magnitude depending on subclass as well as allotype. IgG1 was found to possess exceptionally strong CH3-CH3 interactions. Surprisingly, the Fc parts of all other subclasses dissociate relatively easily. Without properly formed hinge disulfide bonds, IgG2 and IgG3 would be able to participate in Fab arm exchange similarly as IgG4 *in vivo*. Because antibody molecules have a long half-life *in vivo*, they need to be resistant to degradation and structural alterations for prolonged periods of time. In this light the relatively facile dissociation of IgG2 and IgG3 CH3 domains is unexpected. However, the higher number of disulfide bonds in the hinge regions of these subclasses (Fig. 5*C*) acts to counterbalance the overall instability. These results highlight the importance of the hinge disulfide bonds in maintaining the structural integrity of IgG2 and IgG3 molecules. Furthermore, these results imply that for any therapeutic antibody, but in particular IgG4 antibodies with a stabilized hinge (S228P) and bispecific antibodies, it is important to ensure proper formation of hinge disulfide bonds. This is not necessarily the case (13), and even antibodies isolated from serum sometimes contain detectable hinge sulfhydryl groups (59). Conversely, several attempts have been made to produce monovalent antibody fragments by destabilizing CH3 interactions (5, 60). The combination of Asn-392, Met-397, and Arg-409 results in a fragment with weak interactions that will be essentially monomeric at 1 μg/ml using only amino acid substitutions already present individually among the IgG subclasses, thus avoiding the introduction of foreign T- or B-cell epitopes other than allotypic variants. Such monovalent antibody fragments have an increased half-life compared with Fab fragments and can be useful as a therapeutic agent.

Substantial genetic variation exists between individuals in the IgG3 (IGHG3) gene. Here we demonstrate that these polymorphisms lead to variations in the strength of inter-heavy chain interactions in IgG3 by approximately 6 orders of magnitude. Interestingly, one strategy to enhance the potency of therapeutic antibodies for (tumor) cytotoxicity involves the introduction of IgG3 features into an IgG1 backbone or the use of complete IgG3 allotypic variants with extended half-life (61). Natsume *et al.* (62, 63) reported IgG1/IgG3 hybrids with enhanced complement-dependent cytotoxicity compared with IgG1. Although complement-dependent cytotoxicity is initiated by C1q binding to the CH2 domains of IgG, determinants in the CH3 domains of IgG3, tentatively Met-397 (62, 63), were found to also modulate the complement-dependent cytotoxicity efficiency, particularly at low antigen densities. One might speculate that weaker interactions between the CH3 domains causes increased flexibility in the molecule, allowing optimal C1q binding. It would be worthwhile to further investigate the role of CH3 determinants in modulating antibody effector functions.

Stability may suffer from weak inter-heavy chain interactions, as was found for IgG4 (64). On the other hand, the study of Chennamsetty *et al.* (65) identified Val-397 to be part of an aggregation-prone motif in IgG. For this motif to participate in interactions with other IgG molecules, it is likely that dissociation of the CH3 domains has to take place first. Thus, whether or not stabilization of IgG2 or IgG3 with a M397V mutation would affect the aggregation propensity in a positive or negative manner remains to be determined. Interestingly, heavy chain dissociation appears to be a critical step in some forms of aggregation, and the addition of IgG2 CH3 dimers was found to diminish acid-induced aggregation of a therapeutic immunoglobulin formulation, probably by acting as a scaffold (15).

Crystal structure analysis revealed Met-397 to have a weakening effect on the CH3-CH3 interface by increasing interatomic distances for neighboring residues, whereas certain (simulated) Asn-392 conformations would cause steric clashes or even disrupt the conserved water molecule network. From a recent study it is known that Arg-409 adopts two conformations at the IgG4 CH3-CH3 interface, one of which is compatible with a conserved water molecule network, whereas the other is not, resulting in reduced hydrogen bonding and a lowering of the buried surface area (54). Perhaps Asn-392 behaves in a similar manner at the IgG3 CH3-CH3 interface, adopting different conformations, each with a different effect. The 2-fold nature of the CH3-CH3 interface is such that residues 392 and 409 from one chain together with residue 397′ from the other compose one site at each end of the interface, and at each site the Cα atoms for these residues are within 11 Å of one another. This spatial orientation may explain the observation that the enthalpic and entropic contributions of Asn-392, Met-397, and Arg-409 to the overall stability are non-additive.

Sequence variation at position 397 is not unique to human IgG subclasses. For example, residue 397 is valine in murine IgG2a and -b but isoleucine in IgG1 and IgG3. Analysis of the available crystal structures of murine IgG1, IgG2a, and IgG2b (8, 66–68) did not reveal significant changes to interatomic distances at the CH3-CH3 interface. However, whereas D-strand residues 395 and 396 are Pro/Pro in all human IgG subclasses, these are Gln/Pro, Glu/Pro, Ala/Pro, and Pro/Pro in murine IgG1, IgG2a, IgG2b, and IgG3, respectively. Where these residues are Pro/Pro (human IgG and murine IgG3), greater structural rigidity might be expected. Intriguingly, murine IgG3 was found to participate in Fab-arm exchange (69). One could hypothesize that a reduced ability of the CH3-CH3 interface to accommodate the V397I substitution facilitates Fab arm exchange.

At mildly reducing conditions, the rate of Fab arm exchange of IgG4 with a “stabilized” hinge (IgG4-S228P) depends markedly differently on the GSH concentration in comparison to wild-type IgG4; for IgG4-S228P the dependence is approximately *k* ∼ [GSH]^2^, because two disulfide bonds need to be broken simultaneously before dissociation is possible; for IgG4 the rate changes ∼4-fold upon going from 0.5 to 5 mm GSH, reflecting that the major non-covalent isomer being formed is the intrachain form (in which no disulfide bonds are broken, inter-conversion between inter-chain and intra-chain is catalyzed by GSH). The steep concentration dependence for stabilized hinge antibodies explains why on the one hand *in vivo* Fab arm exchange is not observed anymore (57, 58), whereas at the same time *in vitro* Fab arm exchange is conveniently carried out at modest concentrations of reducing agent, *e.g.* to generate bispecific antibodies (Duobodies) (27). The mild reducing conditions used in the present study are not expected to affect the intradomain disulfide bonds. Using hingeless IgG4 Fc fragments, we found the rate of exchange to be unaffected by DTT or GSH up to 10 mm, but above ∼20 mm a noticeable effect was observed (not shown).

In summary, we investigated the dynamics of inter-heavy chain interactions of human subclasses and allotypes using real-time monitoring of Fab arm exchange. We identified structural variation between human IgG subclasses and allotypes at three amino acid positions (Lys/Asn-392, Val/Met-397, Lys/Arg-409) to alter the interdomain interactions of CH3 by >6 orders of magnitude. These interactions are only sufficiently strong for IgG1 to maintain the structural integrity of the molecule during its *in vivo* life-span; for IgG2 and IgG3 the interheavy chain disulfide bonds are essential to prevent half-molecule dissociation, whereas for IgG4 the labile hinge disulfide bonds favor half-molecule exchange.

## Supplementary Material

Supplemental Data

## Footnotes

3 Braden, B. C. 10.2210/pdb3do3/pdb.

4 Moiani, D., Salvalaglio, M., Cavalotti, C., Bujacz, A. D., Redzynia, I., Bujacz, G. D., Dinon, F., Pengo, P., and Fassina, G. 10.2210/pdb3d6g/pdb.

5 Raju, T. S., Mulkerrin, M. G., Parker, M., De Vos, A. M., Gazzano-Santoro, H., Totpal, K., and Ultsch, M. H. 10.2210/pdb1oqo/pdb.
