# Supplementary material for: Dynamics of Inter-heavy Chain Interactions in Human Immunoglobulin G (IgG) Subclasses Studied by Kinetic Fab Arm Exchange
Source: J Biol Chem. 2014 Jan 14;289(9):6098–109. doi: 10.1074/jbc.M113.541813 (PMC3937676; doi:10.1074/jbc.M113.541813)
Supplement: Supplemental Data [file supp_289_9_6098__index.html]

Dynamics of Inter-heavy Chain Interactions in Human Immunoglobulin G (IgG) Subclasses Studied by Kinetic Fab Arm Exchange — Inter-heavy Chain Interactions in Human IgG Subclasses — Supplemental Data 

# Dynamics of Inter-heavy Chain Interactions in Human Immunoglobulin G (IgG) Subclasses Studied by Kinetic Fab Arm Exchange

## Supplemental Data

**Files in this Data Supplement:**

- Supplemental Figures S1 and S2 (.pdf, 57 KB) - Supplemental Figures S1 and S2: S1. alignment of CH3 domains of polymorphisms in human IgG subclasses S2. complete sequences of Fc constructs used in study
