# Supplementary material for: Dynamics of Inter-heavy Chain Interactions in Human Immunoglobulin G (IgG) Subclasses Studied by Kinetic Fab Arm Exchange
Source: J Biol Chem. 2014 Jan 14;289(9):6098–109. doi: 10.1074/jbc.M113.541813 (PMC3937676; doi:10.1074/jbc.M113.541813)
Supplement: Supplemental Data [file supp_M113.541813_jbc.M113.541813-1.pdf]

**CH3**		350|	360|	370|	380|	390|	400|	410|	420|	430|	440|		
G1m(za)	IGHG1*01	GQPREPQVYT	LPPS**RD**E**L**TK	NQVSLTCLVK	GFYPSDIA**V**E	WES**N**GQPENN	Y**K**TTPP**V**LDS	DGSFFLYS**K**L	TVDKSRWQ**Q**G	N**V**FSCSVMHE	**A**LHN**HY**TQKS	LSLS**P**GK	J00228
G1m(zax)	IGHG1*04	GQPREPQVYT	LPPS**RD**E**L**TK	NQVSLTCLVK	GFYPSDIA**V**E	WES**N**GQPENN	Y**K**TTPP**V**LDS	DGSFFLYS**K**L	TVDKSRWQ**Q**G	N**V**FSCSVMHE	**G**LHN**HY**TQKS	LSLS**P**GK	
G1m(zav)		GQPREPQVYT	LPPS**RD**E**L**TK	NQVSLTCLVK	GFYPSDIA**V**E	WES**N**GQPENN	Y**K**TTPP**V**LDS	DGSFFLYS**K**L	TVDKSRWQ**Q**G	N**I**FSCSVMHE	**A**LHN**HY**TQKS	LSLS**P**GK	
G1m(f)	IGHG1*03	GQPREPQVYT	LPPS**RE**E**M**TK	NQVSLTCLVK	GFYPSDIA**V**E	WES**N**GQPENN	Y**K**TTPP**V**LDS	DGSFFLYS**K**L	TVDKSRWQ**Q**G	N**V**FSCSVMHE	**A**LHN**HY**TQKS	LSLS**P**GK	Y14737
G1m(fa)		GQPREPQVYT	LPPS**RD**E**L**TK	NQVSLTCLVK	GFYPSDIA**V**E	WES**N**GQPENN	Y**K**TTPP**V**LDS	DGSFFLYS**K**L	TVDKSRWQ**Q**G	N**V**FSCSVMHE	**A**LHN**HY**TQKS	LSLS**P**GK	
G2m(n)	IGHG2*02	GQPREPQVYT	LPPS**RE**E**M**TK	NQVSLTCLVK	GFYPSDIA**V**E	WES**N**GQPENN	Y**K**TTPP**M**LDS	DGSFFLYS**K**L	TVDKSRWQ**Q**G	N**V**FSCSVMHE	**A**LHN**HY**TQKS	LSLS**P**GK	J00230
G2m(..)	IGHG2*01	GQPREPQVYT	LPPS**RE**E**M**TK	NQVSLTCLVK	GFYPSDIA**V**E	WES**N**GQPENN	Y**K**TTPP**M**LDS	DGSFFLYS**K**L	TVDKSRWQ**Q**G	N**V**FSCSVMHE	**A**LHN**HY**TQKS	LSLS**P**GK	AJ250170
G2m(ny)	IGHG2*04	GQPREPQVYT	LPPS**RE**E**M**TK	NQVSLTCLVK	GFYPSDIA**V**E	WES**N**GQPENN	Y**K**TTPP**M**LDS	DGSFFLYS**K**L	TVDKSRWQ**Q**G	N**V**FSCSVMHE	**A**LHN**HY**TQKS	LSLS**P**GK	AF449617
G3m(b*)	IGHG3*01	GQPREPQVYT	LPPS**RE**E**M**TK	NQVSLTCLVK	GFYPSDIA**V**E	WES**S**GQPENN	Y**N**TTPP**M**LDS	DGSFFLYS**K**L	TVDKSRWQ**Q**G	N**I**FSCSVMHE	**A**LHN**RF**TQKS	LSLS**P**GK	X03604
G3m(b*)	IGHG3*04	GQPREPQVYT	LPPS**RE**E**M**TK	NQVSLTCLVK	GFYPSDIA**V**E	WES**S**GQPENN	Y**N**TTPP**M**LDS	DGSFFLYS**K**L	TVDKSRWQ**Q**G	N**I**FSCSVMHE	**A**LHN**RF**TQKS	LSLS**P**GK	X99549
G3m(b*)	IGHG3*06	GQPREPQVYT	LPPS**RE**E**M**TK	NQVSLTCLVK	GFYPSDIA**V**E	WES**S**GQPENN	Y**K**TTPP**M**LDS	DGSFFLYS**K**L	TVDKSRWQ**Q**G	N**I**FSCSVMHE	**A**LHN**RF**TQKS	LSLS**P**GK	AJ390237
G3m(b*)	IGHG3*09	GQPREPQVYT	LPPS**RE**E**M**TK	NQVSLTCLVK	GFYPSDIA**V**E	WES**S**GQPENN	Y**N**TTPP**M**LDS	DGSFFLYS**K**L	TVDKSRWQ**Q**G	N**I**FSCSVMHE	**A**LHN**RF**TQKS	LSLS**P**GK	AJ390242
G3m(b*)	IGHG3*11	GQPREPQVYT	LPPS**RE**E**M**TK	NQVSLTCLVK	GFYPSDIA**V**E	WES**S**GQPENN	Y**N**TTPP**M**LDS	DGSFFLYS**K**L	TVDKSRWQ**Q**G	N**I**FSCSVMHE	**A**LHN**RF**TQKS	LSLS**P**GK	AJ390247
G3m(b*)	IGHG3*12	GQPREPQVYT	LPPS**RE**E**M**TK	NQVSLTCLVK	GFYPSDIA**V**E	WES**S**GQPENN	Y**N**TTPP**M**LDS	DGSFFLYS**K**L	TVDKSRWQ**Q**G	N**I**FSCSVMHE	**A**LHN**RF**TQKS	LSLS**P**GK	AJ390252
G3m(b**)	IGHG3*08	GQPREPQVYT	LPPS**RE**E**M**TK	NQVSLTCLVK	GFYPSDIA**V**E	WES**N**GQPENN	Y**N**TTPP**M**LDS	DGSFFLYS**K**L	TVDKSRWQ**Q**G	N**I**FSCSVMHE	**A**LHN**RF**TQKS	LSLS**P**GK	AJ390241
G3m(c3c5*)	IGHG3*03	GQPREPQVYT	LPPS**RE**E**M**TK	NQVSLTCLVK	GFYPSDIA**V**E	WES**S**GQPENN	Y**N**TTPP**V**LDS	DGSFFLYS**R**L	TVDKSRWQ**E**G	N**V**FSCSVMHE	**A**LHN**RF**TQKS	LSLS**P**GK	X16110
G3m(c3*)	IGHG3*13	GQPREPQVYT	LPPS**RE**E**M**TK	NQVSLTCLVK	GFYPSDIA**V**E	WES**S**GQPENN	Y**K**TTPP**M**LDS	DGSFFLYS**K**L	TVDKSRWQ**E**G	N**I**FSCSVMHE	**A**LHN**RF**TQKS	LSLS**P**GK	AJ390244
G3m(g*)	IGHG3*14	GQPREPQVYT	LPPS**RE**E**M**TK	NQVSLTCLVK	GFYPSDIA**V**E	WES**N**GQPENN	Y**N**TTPP**M**LDS	DGSFFLYS**K**L	TVDKSRWQ**Q**G	N**I**FSCSVMHE	**A**LHN**RY**TQKS	LSLS**P**GK	AJ390254
G3m(g*)	IGHG3*15	GQPREPQVYT	LPPS**RE**E**M**TK	NQVSLTCLVK	GFYPSDIA**V**E	WES**N**GQPENN	Y**K**TTPP**M**LDS	DGSFFLYS**K**L	TVDKSRWQ**Q**G	N**I**FSCSVMHE	**A**LHN**RY**TQKS	LSLS**P**GK	AJ390260
G3m(g*)	IGHG3*16	GQPREPQVYT	LPPS**RE**E**M**TK	NQVSLTCLVK	GFYPSDIA**V**E	WES**N**GQPENN	Y**N**TTPP**M**LDS	DGSFFLYS**K**L	TVDKSRWQ**Q**G	N**I**FSCSVMHE	**A**LHN**RY**TQKS	LSLS**P**GK	AJ390262
G3m(s*)	IGHG3*17	GQPREPQVYT	LPPS**RE**E**M**TK	NQVSLTCLVK	GFYPSDIA**M**E	WES**S**GQPENN	Y**K**TTPP**V**LDS	DGSFFLYS**K**L	TVDKSRWQ**Q**G	N**I**FSCSVMHE	**A**LHN**HY**TQKS	LSLS**P**GK	AJ390272
G3m(st*)	IGHG3*18	GQPREPQVYT	LPPS**RE**E**M**TK	NQVSLTCLVK	GFYPSDIA**M**E	WES**S**GQPENN	Y**K**TTPP**V**LDS	DGSFFLYS**K**L	TVDKSRWQ**Q**G	N**I**FSCSVMHE	**A**LHN**HY**TQKS	LSLS**P**GK	AJ390276
G3m(st*)	IGHG3*19	GQPREPQVYT	LPPS**RE**E**M**TK	NQVSLTCLVK	GFYPSDIA**M**E	WES**S**GQPENN	Y**K**TTPP**V**LDS	DGSFFLYS**K**L	TVDKSRWQ**Q**G	N**I**FSCSVMHE	**A**LHN**HY**TQKS	LSLS**P**GK	AJ390279
G4m a	IGHG4*01	GQPREPQVYT	LPPS**QE**E**M**TK	NQVSLTCLVK	GFYPSDIA**V**E	WES**N**GQPENN	Y**K**TTPP**V**LDS	DGSFFLYS**R**L	TVDKSRWQ**E**G	N**V**FSCSVMHE	**A**LHN**HY**TQKS	LSLS**L**GK	K01316
G4m a	IGHG4*03	GQPREPQVYT	LPPS**QE**E**M**TK	NQVSLTCLVK	GFYPSDIA**V**E	WES**N**GQPENN	Y**K**TTPP**V**LDS	DGSFFLYS**K**L	TVDKSRWQ**E**G	N**V**FSCSVMHE	**A**LHN**HY**TQKS	LSLS**L**GK	AJ001564
G4m b	IGHG4*02	GQPREPQVYT	LPPS**QE**E**M**TK	NQVSLTCLVK	GFYPSDIA**V**E	WES**N**GQPENN	Y**K**TTPP**V**LDS	DGSFFLYS**R**L	TVDKSRWQ**E**G	N**V**FSCSVMHE	**A**LHN**HY**TQKS	LSLS**L**GK	AJ001563

G3m shorthand notations:

b* = b0,b1,b3,b4,b5,u,v
b** = b1,b4,u,v
c3* = b0,b1,b4,c3,b5,u,v
c3c5* = b0,b1,c3,u,c5
s* = b0,b3,b5,s,v
st* = b0,b3,b5,s,t,v
g* = g1,g5,u,v

```
HINGE                       220|      230|
                            ESK YGPPCPSCP


CH2                                240|       250|       260|       270|       280|       290|       300|       310|       320|       330|       340|
IgG1            IGHG1*01 APELLGGPSV FLFPPKPKDT LMISRTPEVT CVVVDVSHED PEVKFNWYVD GVEVHNAKTK PREEQYNSTY RVVSVLTVLH QDWLNGKEYK CKVSNKALPA PIEKTISKAK
IgG2            IGHG1*01 APPVA-GPSV FLFPPKPKDT LMISRTPEVT CVVVDVSHED PEVQFNWYVD GVEVHNAKTK PREEQFNSTF RVVSVLTVVH QDWLNGKEYK CKVSNKGLPA PIEKTISKTK
G3*01           IGHG3*01 APELLGGPSV FLFPPKPKDT LMISRTPEVT CVVVDVSHED PEVQFKWYVD GVEVHNAKTK PREEQYNSTF RVVSVLTVLH QDWLNGKEYK CKVSNKALPA PIEKTISKTK
G3*06           IGHG3*06 APELLGGPSV FLFPPKPKDT LMISRTPEVT CVVVDVSHED PEVQFKWYVD GVEVHNAKTK PREEQYNSTF RVVSVLTVLH QDWLNGKEYK CKVSNKALPA PIEKTISKTK
G3*03           IGHG3*03 APELLGGPSV FLFPPKPKDT LMISRTPEVT CVVVDVSHED PEVQFKWYVD GVEVHNAKTK PREEQYNSTF RVVSVLTVLH QDWLNGKEYK CKVSNKALPA PIEKTISKTK
G3*17           IGHG3*17 APELLGGPSV FLFPPKPKDT LMISRTPEVT CVVVDVSHED PEVQFKWYVD GVEVHNAKTK PREEQYNSTF RVVSVLTVLH QDWLNGKEYK CKVSNKALPA PIEKTISKTK
IgG4            IGHG4*01 APEFLGGPSV FLFPPKPKDT LMISRTPEVT CVVVDVSQED PEVQFNWYVD GVEVHNAKTK PREEQFNSTY RVVSVLTVLH QDWLNGKEYK CKVSNKGLPS SIEKTISKAK

G1-V397M                 APELLGGPSV FLFPPKPKDT LMISRTPEVT CVVVDVSHED PEVKFNWYVD GVEVHNAKTK PREEQYNSTY RVVSVLTVLH QDWLNGKEYK CKVSNKALPA PIEKTISKAK
G2-M397V                 APPVA-GPSV FLFPPKPKDT LMISRTPEVT CVVVDVSHED PEVQFNWYVD GVEVHNAKTK PREEQFNSTF RVVSVLTVVH QDWLNGKEYK CKVSNKGLPA PIEKTISKTK
G1-K409R                 APELLGGPSV FLFPPKPKDT LMISRTPEVT CVVVDVSHED PEVKFNWYVD GVEVHNAKTK PREEQYNSTY RVVSVLTVLH QDWLNGKEYK CKVSNKALPA PIEKTISKAK
G4-R409K                 APEFLGGPSV FLFPPKPKDT LMISRTPEVT CVVVDVSQED PEVQFNWYVD GVEVHNAKTK PREEQFNSTY RVVSVLTVLH QDWLNGKEYK CKVSNKGLPS SIEKTISKAK
G4-V397M                 APEFLGGPSV FLFPPKPKDT LMISRTPEVT CVVVDVSQED PEVQFNWYVD GVEVHNAKTK PREEQFNSTY RVVSVLTVLH QDWLNGKEYK CKVSNKGLPS SIEKTISKAK
G4-K392NV397M            APEFLGGPSV FLFPPKPKDT LMISRTPEVT CVVVDVSQED PEVQFNWYVD GVEVHNAKTK PREEQFNSTY RVVSVLTVLH QDWLNGKEYK CKVSNKGLPS SIEKTISKAK


CH3                                350|       360|       370|       380|       390|       400|       410|       420|       430|       440|
IgG1            IGHG1*01 GQPREPQVYT LPPSRDELTK NQVSLTCLVK GFYPSDIAVE WESNGQPENN YKTTPPVLDS DGSFFLYSKL TVDKSRWQQG NVFSCSVMHE ALHNHYTQKS LSLSPGK
IgG2            IGHG2*01 GQPREPQVYT LPPSREEMTK NQVSLTCLVK GFYPSDIAVE WESNGQPENN YKTTPPMLDS DGSFFLYSKL TVDKSRWQQG NVFSCSVMHE ALHNHYTQKS LSLSPGK
G3*01           IGHG3*01 GQPREPQVYT LPPSREEMTK NQVSLTCLVK GFYPSDIAVE WESSGQPENN YNTTPPMLDS DGSFFLYSKL TVDKSRWQQG NIFSCSVMHE ALHNRFTQKS LSLSPGK
G3*06           IGHG3*06 GQPREPQVYT LPPSREEMTK NQVSLTCLVK GFYPSDIAVE WESSGQPENN YKTTPPMLDS DGSFFLYSKL TVDKSRWQQG NIFSCSVMHE ALHNRFTQKS LSLSPGK
G3*03           IGHG3*03 GQPREPQVYT LPPSREEMTK NQVSLTCLVK GFYPSDIAVE WESSGQPENN YNTTPPVLDS DGSFFLYSRL TVDKSRWQEG NVFSCSVMHE ALHNRFTQKS LSLSPGK
G3*17           IGHG3*17 GQPREPQVYT LPPSREEMTK NQVSLTCLVK GFYPSDIAME WESSGQPENN YKTTPPVLDS DGSFFLYSKL TVDKSRWQQG NIFSCSVMHE ALHNHYTQKS LSLSPGK
IgG4            IGHG4*01 GQPREPQVYT LPPSQEEMTK NQVSLTCLVK GFYPSDIAVE WESNGQPENN YKTTPPVLDS DGSFFLYSRL TVDKSRWQEG NVFSCSVMHE ALHNHYTQKS LSLSLGK

G1-V397M                 GQPREPQVYT LPPSRDELTK NQVSLTCLVK GFYPSDIAVE WESNGQPENN YKTTPPMLDS DGSFFLYSKL TVDKSRWQQG NVFSCSVMHE ALHNHYTQKS LSLSPGK
G2-M397V                 GQPREPQVYT LPPSREEMTK NQVSLTCLVK GFYPSDIAVE WESNGQPENN YKTTPPVLDS DGSFFLYSKL TVDKSRWQQG NVFSCSVMHE ALHNHYTQKS LSLSPGK
G1-K409R                 GQPREPQVYT LPPSRDELTK NQVSLTCLVK GFYPSDIAVE WESNGQPENN YKTTPPVLDS DGSFFLYSRL TVDKSRWQQG NVFSCSVMHE ALHNHYTQKS LSLSPGK
G4-R409K                 GQPREPQVYT LPPSQEEMTK NQVSLTCLVK GFYPSDIAVE WESNGQPENN YKTTPPVLDS DGSFFLYSKL TVDKSRWQEG NVFSCSVMHE ALHNHYTQKS LSLSLGK
G4-V397M                 GQPREPQVYT LPPSQEEMTK NQVSLTCLVK GFYPSDIAVE WESNGQPENN YKTTPPMLDS DGSFFLYSRL TVDKSRWQEG NVFSCSVMHE ALHNHYTQKS LSLSLGK
G4-K392NV397M            GQPREPQVYT LPPSQEEMTK NQVSLTCLVK GFYPSDIAVE WESNGQPENN YNTTPPMLDS DGSFFLYSRL TVDKSRWQEG NVFSCSVMHE ALHNHYTQKS LSLSLGK
```
